# Poly[[bis­[μ_2_-8-ethyl-5-oxo-2-(piperazin-1-yl)-5,8-dihydro­pyrido[2,3-*d*]pyrimidine-6-carboxyl­ato]zinc(II)] dihydrate]

**DOI:** 10.1107/S1600536809036939

**Published:** 2009-09-19

**Authors:** Wen Xu, Da-Sheng Zhu, Xiao-Dan Song, Zhe An

**Affiliations:** a Second Hospital, Jilin University, Changchun 130041, People’s Republic of China; b School of Pharmaceutical Science, Harbin Medical University, Harbin, 150086, People’s Republic of China

## Abstract

The title compound, {[Zn(C_14_H_16_N_5_O_3_)_2_]·2H_2_O}_*n*_ or [Zn(ppa)_2_]·2H_2_O}_*n*_, where ppa = 8-ethyl-5,8-dihydro-5-oxo-2-(1-piperazin­yl)-pyrido(2,3-*d*)-pyrimidine-6-carboxyl­ate, was synthesized under hydro­thermal conditions. The Zn^II^ atom (site symmetry 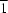) exhibits a distorted *trans*-ZnN_2_O_4_ octa­hedral geometry defined by two monodentate *N*-bonded and two bidentate *O*,*O*-bonded ppa monoanions. The extended two-dimensional structure arising from this connectivity is a square grid and the disordered uncoordinated water mol­ecules occupy cavities within the grid. An N—H⋯O hydrogen bond occurs.

## Related literature

For manganese complexes of the ppa anion, see: Huang *et al.* (2008 ▶). For background to the medicinal uses of pipemidic acid, see: Mizuki *et al.* (1996 ▶).
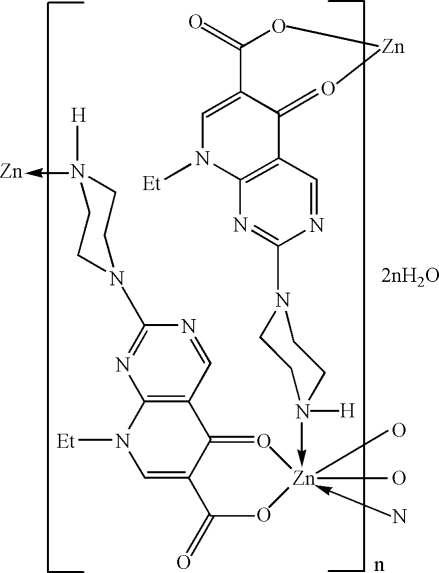


## Experimental

### 

#### Crystal data

[Zn(C_14_H_16_N_5_O_3_)_2_]·2H_2_O
*M* *_r_* = 704.05
Monoclinic, 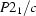

*a* = 6.1146 (12) Å
*b* = 21.424 (4) Å
*c* = 12.577 (3) Å
β = 101.10 (3)°
*V* = 1616.9 (6) Å^3^
*Z* = 2
Mo *K*α radiation
μ = 0.82 mm^−1^
*T* = 295 K
0.36 × 0.28 × 0.18 mm

#### Data collection

Rigaku R-AXIS RAPID diffractometer
Absorption correction: multi-scan (*CrystalStructure*; Rigaku/MSC, 2002 ▶) *T*_min_ = 0.756, *T*_max_ = 0.866
15697 measured reflections
3684 independent reflections
2570 reflections with *I* > 2σ(*I*)
*R*_int_ = 0.045

#### Refinement

*R*[*F* ^2^ > 2σ(*F* ^2^)] = 0.057
*wR*(*F* ^2^) = 0.210
*S* = 1.06
3684 reflections
228 parameters
1 restraint
H atoms treated by a mixture of independent and constrained refinement
Δρ_max_ = 0.83 e Å^−3^
Δρ_min_ = −0.83 e Å^−3^

### 

Data collection: *RAPID-AUTO* (Rigaku, 1998 ▶); cell refinement: *RAPID-AUTO*; data reduction: *CrystalStructure* (Rigaku/MSC, 2002 ▶); program(s) used to solve structure: *SHELXS97* (Sheldrick, 2008 ▶); program(s) used to refine structure: *SHELXL97* (Sheldrick, 2008 ▶); molecular graphics: *ORTEPII* (Johnson, 1976 ▶); software used to prepare material for publication: *SHELXL97*.

## Supplementary Material

Crystal structure: contains datablocks I, global. DOI: 10.1107/S1600536809036939/hb5062sup1.cif

Structure factors: contains datablocks I. DOI: 10.1107/S1600536809036939/hb5062Isup2.hkl

Additional supplementary materials:  crystallographic information; 3D view; checkCIF report

## Figures and Tables

**Table 1:** Selected bond lengths (Å)

Zn1—O1	2.031 (3)
Zn1—O3	2.107 (2)
Zn1—N5^i^	2.275 (3)

Symmetry code: (i) 
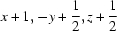
.

**Table 2:** Hydrogen-bond geometry (Å, °)

*D*—H⋯*A*	*D*—H	H⋯*A*	*D*⋯*A*	*D*—H⋯*A*
N5—H5*N*⋯O2^ii^	0.91 (5)	2.28 (5)	3.168 (5)	166 (4)

Symmetry code: (ii) 
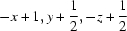
.

**Fig. 1.**
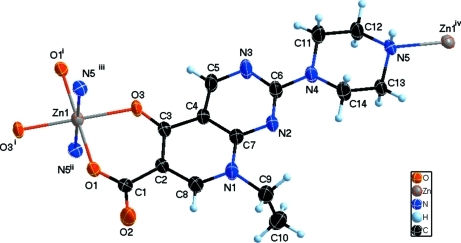
The asymmetric unit of (I) extended to show the zinc coordination sphere showing the showing 50% displacement ellipsoids (water molecule O atoms have been omitted for clarity).

**Fig. 2.**
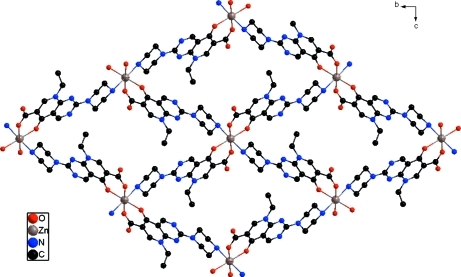
A view of part of a two-dimensional polymeric sheet in (I) showing the square-grid connectivity (H atoms and water molecule O atoms omitted for clarity).

## supplementary crystallographic information

### Comment

Pipemidic acid (Hppa, C_14_H_16_N_5_O_3_, 8-Ethyl-5,8-dihydro-5-oxo-2- (1-piperazinyl)-pyrido(2,3 - d)-pyrimidine-6-carboxylic acid) is member of a class of quinolones used to treat infections (Mizuki *et al.*, 1996). The manganese complex of the ppa anion has been reported (Huang *et al.*, 2008); the title zinc(II) complex is reported here (Fig. 1).

The zinc(II) atom is coordinated by four oxygen atoms and two N atoms from four ppa ligands (two monodentate-N and two O,*O*-bidentate) to form a square grid (Fig. 2). The disordered, uncoordinated, water molecules occupy the cavities.

### Experimental

A mixture of Zn(CH_3_COO)_2_.2H_2_O (0.055 g, 0.25 mmol), Hppa (0.15 g, 0.5 mmol), sodium hydroxide (0.04 g, 1 mmol) and water (12 ml) was stirred for 30 min in air. The mixture was then transferred to a 23 ml Teflon-lined hydrothermal bomb. The bomb was kept at 433 K for 72 h under autogenous pressure. Upon cooling, colourless prisms of (I) were obtained from the reaction mixture.

### Refinement

The carbon-bound H atoms were positioned geometrically (C—H = 0.93–0.97 Å) and were included in the refinement in the riding model approximation, with U_iso_(H) = 1.2U_eq_(C). The H on Nitrogen atoms were located in a difference Fourier map, and were refined with a distance restraint of N—H = 0.86 (1)Å and with *U*_iso_(H) = 1.2U_eq_(N).

The water H atoms could not be placed due to the disorder of the O atoms.

### Crystal data

**Table:**

[Zn(C_14_H_16_N_5_O_3_)_2_]·2H_2_O	*Z* = 2
*M**_r_* = 704.05	*F*(000) = 728
Monoclinic, *P*2_1_/*c*	*D*_x_ = 1.442 Mg m^−^^3^
Hall symbol: -P 2ybc	Mo *K*α radiation, λ = 0.71073 Å
*a* = 6.1146 (12) Å	µ = 0.82 mm^−^^1^
*b* = 21.424 (4) Å	*T* = 295 K
*c* = 12.577 (3) Å	Prism, colorless
β = 101.10 (3)°	0.36 × 0.28 × 0.18 mm
*V* = 1616.9 (6) Å^3^	

### Data collection

**Table:**

Rigaku R-AXIS RAPID diffractometer	3684 independent reflections
Radiation source: fine-focus sealed tube	2570 reflections with *I* > 2σ(*I*)
graphite	*R*_int_ = 0.045
Detector resolution: 10.000 pixels mm^-1^	θ_max_ = 27.5°, θ_min_ = 3.3°
ω scans	*h* = −7→7
Absorption correction: multi-scan (*CrystalStructure*; Rigaku/MSC, 2002)	*k* = −27→25
*T*_min_ = 0.756, *T*_max_ = 0.866	*l* = −16→16
15697 measured reflections	

### Refinement

**Table:**

Refinement on *F*^2^	Primary atom site location: structure-invariant direct methods
Least-squares matrix: full	Secondary atom site location: difference Fourier map
*R*[*F*^2^ > 2σ(*F*^2^)] = 0.057	Hydrogen site location: inferred from neighbouring sites
*wR*(*F*^2^) = 0.210	H atoms treated by a mixture of independent and constrained refinement
*S* = 1.06	*w* = 1/[σ^2^(*F*_o_^2^) + (0.1254*P*)^2^ + 1.7801*P*] where *P* = (*F*_o_^2^ + 2*F*_c_^2^)/3
3684 reflections	(Δ/σ)_max_ < 0.001
228 parameters	Δρ_max_ = 0.83 e Å^−^^3^
1 restraint	Δρ_min_ = −0.83 e Å^−^^3^

### Special details

**Table:**

Geometry. All e.s.d.'s (except the e.s.d. in the dihedral angle between two l.s. planes) are estimated using the full covariance matrix. The cell e.s.d.'s are taken into account individually in the estimation of e.s.d.'s in distances, angles and torsion angles; correlations between e.s.d.'s in cell parameters are only used when they are defined by crystal symmetry. An approximate (isotropic) treatment of cell e.s.d.'s is used for estimating e.s.d.'s involving l.s. planes.
Refinement. Refinement of *F*^2^ against ALL reflections. The weighted *R*-factor *wR* and goodness of fit *S* are based on *F*^2^, conventional *R*-factors *R* are based on *F*, with *F* set to zero for negative *F*^2^. The threshold expression of *F*^2^ > σ(*F*^2^) is used only for calculating *R*-factors(gt) *etc*. and is not relevant to the choice of reflections for refinement. *R*-factors based on *F*^2^ are statistically about twice as large as those based on *F*, and *R*- factors based on ALL data will be even larger.

### Fractional atomic coordinates and isotropic or equivalent isotropic displacement parameters (Å^2^)

**Table:**

	*x*	*y*	*z*	*U*_iso_*/*U*_eq_	Occ. (<1)
O1W	−0.045 (3)	−0.0632 (10)	−0.0746 (11)	0.187 (8)	0.50
O2W	0.340 (5)	0.0205 (10)	−0.0364 (12)	0.251 (14)	0.50
Zn1	0.5000	0.0000	0.5000	0.0265 (2)	
O1	0.6981 (4)	−0.00325 (10)	0.3877 (2)	0.0271 (6)	
O2	0.8573 (7)	0.01818 (18)	0.2500 (3)	0.0616 (11)	
O3	0.3495 (5)	0.07935 (11)	0.4179 (2)	0.0317 (6)	
N1	0.4916 (7)	0.17173 (17)	0.1532 (3)	0.0481 (10)	
N2	0.2252 (6)	0.24690 (15)	0.1677 (3)	0.0386 (8)	
N3	−0.0127 (6)	0.23572 (16)	0.2988 (3)	0.0436 (9)	
N4	−0.0227 (6)	0.32384 (15)	0.1907 (3)	0.0349 (8)	
N5	−0.2450 (5)	0.43908 (14)	0.1084 (2)	0.0273 (7)	
H5N	−0.154 (8)	0.466 (2)	0.152 (4)	0.065 (16)*	
C1	0.7147 (7)	0.02891 (17)	0.3064 (3)	0.0316 (8)	
C2	0.5658 (7)	0.08450 (17)	0.2772 (3)	0.0317 (8)	
C3	0.3947 (6)	0.10453 (16)	0.3346 (3)	0.0271 (7)	
C4	0.2744 (7)	0.15974 (16)	0.2910 (3)	0.0303 (8)	
C5	0.0937 (8)	0.18359 (19)	0.3318 (3)	0.0398 (10)	
H5	0.0454	0.1610	0.3861	0.048*	
C6	0.0671 (7)	0.26762 (18)	0.2197 (3)	0.0327 (8)	
C7	0.3246 (7)	0.19340 (18)	0.2034 (3)	0.0360 (9)	
C8	0.6010 (8)	0.1189 (2)	0.1902 (4)	0.0457 (11)	
H8	0.7096	0.1047	0.1535	0.055*	
C9	0.5540 (11)	0.2051 (3)	0.0585 (5)	0.0665 (16)	
H9B	0.5306	0.2496	0.0653	0.080*	
H9A	0.7107	0.1983	0.0582	0.080*	
C10	0.4247 (16)	0.1834 (5)	−0.0401 (7)	0.116 (3)	
H10C	0.4566	0.1401	−0.0496	0.174*	
H10B	0.4605	0.2071	−0.0993	0.174*	
H10A	0.2693	0.1883	−0.0385	0.174*	
C11	−0.1608 (8)	0.3572 (2)	0.2553 (4)	0.0475 (11)	
H11B	−0.0673	0.3839	0.3077	0.057*	
H11A	−0.2346	0.3275	0.2947	0.057*	
C12	−0.3356 (7)	0.3970 (2)	0.1813 (4)	0.0398 (10)	
H12B	−0.4429	0.3694	0.1379	0.048*	
H12A	−0.4150	0.4218	0.2262	0.048*	
C13	−0.1090 (6)	0.40176 (17)	0.0469 (3)	0.0317 (8)	
H13A	−0.0405	0.4295	0.0018	0.038*	
H13B	−0.2057	0.3734	−0.0006	0.038*	
C14	0.0708 (7)	0.36460 (18)	0.1185 (4)	0.0369 (9)	
H14B	0.1500	0.3397	0.0737	0.044*	
H14A	0.1768	0.3929	0.1610	0.044*	

### Atomic displacement parameters (Å^2^)

**Table:**

	*U*^11^	*U*^22^	*U*^33^	*U*^12^	*U*^13^	*U*^23^
O1W	0.209 (18)	0.28 (2)	0.094 (9)	−0.004 (16)	0.091 (11)	0.013 (12)
O2W	0.47 (4)	0.202 (17)	0.077 (9)	0.11 (2)	0.049 (16)	−0.057 (12)
Zn1	0.0317 (4)	0.0194 (3)	0.0294 (3)	0.0006 (2)	0.0079 (2)	0.0028 (2)
O1	0.0276 (13)	0.0218 (12)	0.0333 (14)	0.0019 (9)	0.0089 (10)	0.0029 (10)
O2	0.073 (3)	0.062 (2)	0.060 (2)	0.040 (2)	0.0387 (19)	0.0275 (18)
O3	0.0378 (15)	0.0223 (12)	0.0368 (14)	0.0092 (10)	0.0116 (11)	0.0121 (11)
N1	0.065 (3)	0.0398 (19)	0.046 (2)	0.0223 (18)	0.0265 (19)	0.0195 (17)
N2	0.051 (2)	0.0282 (16)	0.0400 (18)	0.0135 (15)	0.0160 (16)	0.0124 (14)
N3	0.044 (2)	0.0361 (18)	0.055 (2)	0.0166 (16)	0.0219 (17)	0.0231 (17)
N4	0.0390 (19)	0.0283 (16)	0.0415 (18)	0.0096 (14)	0.0177 (15)	0.0119 (14)
N5	0.0265 (16)	0.0219 (14)	0.0324 (15)	0.0064 (12)	0.0028 (12)	0.0034 (13)
C1	0.037 (2)	0.0274 (18)	0.0316 (18)	0.0046 (15)	0.0112 (16)	−0.0002 (16)
C2	0.038 (2)	0.0238 (17)	0.0354 (19)	0.0070 (15)	0.0110 (16)	0.0035 (15)
C3	0.0283 (18)	0.0219 (16)	0.0302 (17)	0.0007 (13)	0.0032 (14)	0.0013 (14)
C4	0.036 (2)	0.0242 (17)	0.0318 (18)	0.0021 (15)	0.0087 (15)	0.0055 (15)
C5	0.048 (3)	0.033 (2)	0.043 (2)	0.0088 (18)	0.0188 (19)	0.0174 (18)
C6	0.033 (2)	0.0273 (18)	0.0380 (19)	0.0046 (15)	0.0078 (16)	0.0077 (16)
C7	0.044 (2)	0.0323 (19)	0.0347 (19)	0.0092 (17)	0.0137 (17)	0.0067 (17)
C8	0.060 (3)	0.037 (2)	0.046 (2)	0.018 (2)	0.026 (2)	0.0107 (19)
C9	0.083 (4)	0.063 (3)	0.062 (3)	0.030 (3)	0.038 (3)	0.024 (3)
C10	0.117 (7)	0.144 (9)	0.090 (5)	0.031 (6)	0.028 (5)	0.024 (6)
C11	0.055 (3)	0.045 (2)	0.047 (2)	0.025 (2)	0.021 (2)	0.020 (2)
C12	0.040 (2)	0.036 (2)	0.047 (2)	0.0157 (17)	0.0179 (18)	0.0161 (19)
C13	0.035 (2)	0.0247 (17)	0.0366 (19)	0.0101 (15)	0.0094 (16)	0.0081 (15)
C14	0.033 (2)	0.0293 (18)	0.051 (2)	0.0043 (16)	0.0147 (17)	0.0119 (18)

### Geometric parameters (Å, °)

**Table:**

Zn1—O1	2.031 (3)	C2—C8	1.370 (6)
Zn1—O1^i^	2.031 (3)	C2—C3	1.446 (5)
Zn1—O3^i^	2.107 (2)	C3—C4	1.444 (5)
Zn1—O3	2.107 (2)	C4—C7	1.399 (5)
Zn1—N5^ii^	2.275 (3)	C4—C5	1.401 (6)
Zn1—N5^iii^	2.275 (3)	C5—H5	0.9300
O1—C1	1.253 (5)	C8—H8	0.9300
O2—C1	1.247 (5)	C9—C10	1.415 (11)
O3—C3	1.256 (4)	C9—H9B	0.9700
N1—C8	1.351 (5)	C9—H9A	0.9700
N1—C7	1.381 (5)	C10—H10C	0.9600
N1—C9	1.500 (6)	C10—H10B	0.9600
N2—C7	1.334 (5)	C10—H10A	0.9600
N2—C6	1.343 (5)	C11—C12	1.534 (5)
N3—C5	1.319 (5)	C11—H11B	0.9700
N3—C6	1.373 (5)	C11—H11A	0.9700
N4—C6	1.345 (5)	C12—H12B	0.9700
N4—C14	1.454 (5)	C12—H12A	0.9700
N4—C11	1.466 (5)	C13—C14	1.508 (5)
N5—C12	1.468 (5)	C13—H13A	0.9700
N5—C13	1.475 (5)	C13—H13B	0.9700
N5—Zn1^iv^	2.275 (3)	C14—H14B	0.9700
N5—H5N	0.900 (10)	C14—H14A	0.9700
C1—C2	1.501 (5)		
			
O1—Zn1—O1^i^	180.0	N2—C6—N4	117.3 (3)
O1—Zn1—O3^i^	92.90 (10)	N2—C6—N3	125.3 (4)
O1^i^—Zn1—O3^i^	87.10 (10)	N4—C6—N3	117.3 (4)
O1—Zn1—O3	87.10 (10)	N2—C7—N1	117.6 (3)
O1^i^—Zn1—O3	92.90 (10)	N2—C7—C4	123.6 (4)
O3^i^—Zn1—O3	180.0	N1—C7—C4	118.7 (3)
O1—Zn1—N5^ii^	89.74 (11)	N1—C8—C2	125.6 (4)
O1^i^—Zn1—N5^ii^	90.26 (11)	N1—C8—H8	117.2
O3^i^—Zn1—N5^ii^	90.86 (11)	C2—C8—H8	117.2
O3—Zn1—N5^ii^	89.14 (11)	C10—C9—N1	110.8 (7)
O1—Zn1—N5^iii^	90.26 (11)	C10—C9—H9B	109.5
O1^i^—Zn1—N5^iii^	89.74 (11)	N1—C9—H9B	109.5
O3^i^—Zn1—N5^iii^	89.14 (11)	C10—C9—H9A	109.5
O3—Zn1—N5^iii^	90.86 (11)	N1—C9—H9A	109.5
N5^ii^—Zn1—N5^iii^	180.0	H9B—C9—H9A	108.1
C1—O1—Zn1	134.5 (2)	C9—C10—H10C	109.5
C3—O3—Zn1	127.6 (2)	C9—C10—H10B	109.5
C8—N1—C7	119.0 (3)	H10C—C10—H10B	109.5
C8—N1—C9	119.2 (4)	C9—C10—H10A	109.5
C7—N1—C9	121.8 (4)	H10C—C10—H10A	109.5
C7—N2—C6	116.3 (3)	H10B—C10—H10A	109.5
C5—N3—C6	115.3 (4)	N4—C11—C12	110.0 (3)
C6—N4—C14	121.2 (3)	N4—C11—H11B	109.7
C6—N4—C11	122.4 (3)	C12—C11—H11B	109.7
C14—N4—C11	113.0 (3)	N4—C11—H11A	109.7
C12—N5—C13	108.3 (3)	C12—C11—H11A	109.7
C12—N5—Zn1^iv^	115.4 (2)	H11B—C11—H11A	108.2
C13—N5—Zn1^iv^	112.8 (2)	N5—C12—C11	114.7 (3)
C12—N5—H5N	106 (4)	N5—C12—H12B	108.6
C13—N5—H5N	108 (4)	C11—C12—H12B	108.6
Zn1^iv^—N5—H5N	106 (4)	N5—C12—H12A	108.6
O2—C1—O1	122.6 (4)	C11—C12—H12A	108.6
O2—C1—C2	117.7 (3)	H12B—C12—H12A	107.6
O1—C1—C2	119.7 (3)	N5—C13—C14	113.1 (3)
C8—C2—C3	118.6 (3)	N5—C13—H13A	109.0
C8—C2—C1	116.2 (3)	C14—C13—H13A	109.0
C3—C2—C1	125.2 (3)	N5—C13—H13B	109.0
O3—C3—C4	119.4 (3)	C14—C13—H13B	109.0
O3—C3—C2	125.8 (3)	H13A—C13—H13B	107.8
C4—C3—C2	114.7 (3)	N4—C14—C13	111.2 (3)
C7—C4—C5	114.1 (3)	N4—C14—H14B	109.4
C7—C4—C3	123.2 (4)	C13—C14—H14B	109.4
C5—C4—C3	122.6 (3)	N4—C14—H14A	109.4
N3—C5—C4	124.7 (4)	C13—C14—H14A	109.4
N3—C5—H5	117.6	H14B—C14—H14A	108.0
C4—C5—H5	117.6		
			
O1^i^—Zn1—O1—C1	−50 (2)	C11—N4—C6—N2	−167.0 (4)
O3^i^—Zn1—O1—C1	179.5 (4)	C14—N4—C6—N3	171.7 (4)
O3—Zn1—O1—C1	−0.5 (4)	C11—N4—C6—N3	14.0 (6)
N5^ii^—Zn1—O1—C1	88.7 (4)	C5—N3—C6—N2	7.0 (7)
N5^iii^—Zn1—O1—C1	−91.3 (4)	C5—N3—C6—N4	−174.0 (4)
O1—Zn1—O3—C3	0.4 (3)	C6—N2—C7—N1	−178.5 (4)
O1^i^—Zn1—O3—C3	−179.6 (3)	C6—N2—C7—C4	−1.0 (6)
O3^i^—Zn1—O3—C3	65 (100)	C8—N1—C7—N2	177.5 (4)
N5^ii^—Zn1—O3—C3	−89.4 (3)	C9—N1—C7—N2	−2.7 (7)
N5^iii^—Zn1—O3—C3	90.6 (3)	C8—N1—C7—C4	−0.1 (7)
Zn1—O1—C1—O2	178.2 (3)	C9—N1—C7—C4	179.7 (5)
Zn1—O1—C1—C2	1.0 (6)	C5—C4—C7—N2	6.2 (6)
O2—C1—C2—C8	−0.1 (6)	C3—C4—C7—N2	−174.9 (4)
O1—C1—C2—C8	177.1 (4)	C5—C4—C7—N1	−176.3 (4)
O2—C1—C2—C3	−178.8 (4)	C3—C4—C7—N1	2.5 (6)
O1—C1—C2—C3	−1.6 (6)	C7—N1—C8—C2	−1.9 (8)
Zn1—O3—C3—C4	−179.0 (2)	C9—N1—C8—C2	178.3 (5)
Zn1—O3—C3—C2	−1.1 (5)	C3—C2—C8—N1	1.4 (7)
C8—C2—C3—O3	−177.0 (4)	C1—C2—C8—N1	−177.4 (4)
C1—C2—C3—O3	1.7 (6)	C8—N1—C9—C10	90.7 (7)
C8—C2—C3—C4	1.0 (6)	C7—N1—C9—C10	−89.1 (7)
C1—C2—C3—C4	179.7 (4)	C6—N4—C11—C12	−148.9 (4)
O3—C3—C4—C7	175.2 (4)	C14—N4—C11—C12	51.8 (5)
C2—C3—C4—C7	−2.9 (6)	C13—N5—C12—C11	53.7 (5)
O3—C3—C4—C5	−6.0 (6)	Zn1^iv^—N5—C12—C11	−178.8 (3)
C2—C3—C4—C5	175.9 (4)	N4—C11—C12—N5	−52.7 (5)
C6—N3—C5—C4	−0.8 (7)	C12—N5—C13—C14	−55.0 (4)
C7—C4—C5—N3	−5.2 (7)	Zn1^iv^—N5—C13—C14	176.0 (2)
C3—C4—C5—N3	175.9 (4)	C6—N4—C14—C13	145.9 (4)
C7—N2—C6—N4	174.8 (4)	C11—N4—C14—C13	−54.5 (5)
C7—N2—C6—N3	−6.2 (7)	N5—C13—C14—N4	56.5 (5)
C14—N4—C6—N2	−9.3 (6)		

Symmetry codes: (i) −*x*+1, −*y*, −*z*+1; (ii) −*x*, *y*−1/2, −*z*+1/2; (iii) *x*+1, −*y*+1/2, *z*+1/2; (iv) −*x*, *y*+1/2, −*z*+1/2.

### Hydrogen-bond geometry (Å, °)

**Table:**

*D*—H···*A*	*D*—H	H···*A*	*D*···*A*	*D*—H···*A*
N5—H5N···O2^v^	0.91 (5)	2.28 (5)	3.168 (5)	166 (4)

Symmetry codes: (v) −*x*+1, *y*+1/2, −*z*+1/2.
